# Extraction and Characterization of Cellulose from Broccoli Stems as a New Biopolymer Source for Producing Carboxymethyl Cellulose Films

**DOI:** 10.1155/2024/7661288

**Published:** 2024-04-17

**Authors:** Sara Sayanjali, Yuzhou Lu, Kate Howell

**Affiliations:** School of Agriculture, Food and Ecosystem Sciences, University of Melbourne, Parkville, Victoria 3010, Australia

## Abstract

The use of food and agricultural waste-derived carboxymethyl cellulose (CMC) has become of interest due to their biodegradability and cost-effectiveness. In the current research, cellulose was extracted from broccoli stems to produce carboxymethyl cellulose using a carboxymethylation reaction via chloroacetic acid (CAA) and sodium hydroxide (5-10 M). The effects of different synthesis conditions on the degree of substitution (DS) and viscosity of the synthesized CMC powder were investigated. The mechanical properties, water vapor permeability (WVP), and colour of CMC films were also evaluated. The results showed that CMC with the highest DS value (0.60) and the highest viscosity of 0.5 Pa·s could be synthesized from broccoli stems at a concentration of 7.5 M NaOH and a cellulose-to-chloroacetic acid ratio of 1 : 1.2. At CMC concentration of 4 g/100 mL with 0.8 g/100 mL of glycerol, the films had the highest tensile strength (31.91 MPa), whereas with 1.2 g/100 mL glycerol, more flexible films with elongation at break of 27.56% were produced. CMC films with the highest WVP (7.87 × 10^3^ gm^2^·mmHg^−1^/day) were made with 6 g/100 mL of CMC and 1.8 g/100 mL of glycerol. This research proposes a new source of cellulose to produce biodegradable packaging materials to initiate a practical basis for food waste reuse.

## 1. Introduction

Food waste is a considerable global problem, with over 931 million tonnes of food wasted globally, accounting for almost 17% of global food output [1]. Australia also confronts such a problem, wasting approximately 7.6 million tonnes of food each year from farm to household across the entire supply and consumption chains [2]. According to a recent survey, the top five most wasted foods in Australia are vegetables, bread, fruit, bagged salad, and leftovers [3]. All this results in not only significant financial waste but also large amounts of carbon dioxide released during food production and processing, responsible for the increasingly serious greenhouse effect. Given this situation, it has fuelled a desire to achieve the goal of sustainable development by reusing food waste and discovering bioplastic materials as an alternative to conventional food packaging [4].

Broccoli waste accounts for more than 30% of total vegetable wastes in Australia [5]. From farm to market, nearly 70% of broccoli grown is unsaleable, resulting in a waste problem due to a failure to meet trading standards or a lack of a market [6]. In most households, the stems and leaves are discarded as inedible, which consist of nearly 2/3 of the total weight of the broccoli plants [5]. In fact, these discarded parts are still valuable. Broccoli stems are high in fibre (containing nearly 35% of dry weight dietary fibre), and cellulose fibre not only plays an important role in the human food cycle, but it is also an essential raw material required in modern industries [7]. The insolubility of cellulose in water and most common solvents limits its application [8]. Chemical modification of cellulose to produce cellulose derivatives with good solubility in common solvents, such as cellulose ethers and cellulose esters, compensates for the less desirable properties of cellulose, increases its value, and broadens its versatility [8, 9].

Carboxymethyl cellulose is a promising cellulose derivative and is synthesized from cellulose through alkylation and etherification procedures. CMC has the potential to be utilized in food packaging materials due to excellent film-forming ability and mechanical strength [10]. CMC derived from food waste has emerged as a promising biodegradable material and offers several environmental advantages, such as biocompatibility, biodegradability, and renewability [11]. There are increasing issues concerning the fossil-based plastic application in food packaging. To address the issue, the development and promotion of more sustainable materials such as CMC obtained from food waste gained researchers' attention [12]. CMC exhibits varied properties that contribute to the improvement and innovation of food packaging solutions. Its applications include functional coatings for prolonged shelf life [13] and responsive materials capable of adapting to environmental conditions [14]. The application of CMC film coating is one of the most important postharvest practices in the fruit and vegetable industry to maintain the quality of these products due to its moisture retention properties [15]. CMC films can be also used as a carrier for functional bioactive compounds, such as antioxidant [16] and antimicrobial agents [17].

Many researchers have investigated the application of CMC from different sources. Hidayati et al. [18] obtained CMC from seaweed waste to prepare CMC films and explored their biodegradability. The results showed that the biodegradable CMC films decomposed after 14 days, which was more environmentally friendly than plastic packaging. CMC film can also be combined with other active ingredients to produce packaging materials with specific properties. Akhtar et al. [19] enriched CMC-based film with chickpea hull polysaccharides and proved that the film has antibacterial and antioxidant properties, which could be utilized as active packaging for improving the shelf life of food products. CMC can be extracted from various cellulosic wastes, especially biomass wastes, such as pineapple peel [20], young palmyra palm fruit husk [21], asparagus stalk [22], and office waste paper [23], and its characteristics including degree of substitution, rheological properties, viscosity, thermal stability, and morphology have been examined. The optimal reaction conditions for preparing CMC films have shown that depending on the original sources, temperature, etherification agent concentration, and reaction time, the properties of CMC film are varied [24–26].

In the current research, we are aiming to explore the effect of NaOH concentrations of CMC synthesized from broccoli stems on the degree of substitution (DS) and viscosity of CMC powder. We investigated the characteristics of CMC films including colour, mechanical properties, and water vapor permeability from broccoli stems and led to the consideration of CMC from food waste as a renewable source for biodegradable film and coating materials.

## 2. Materials and Methods

### 2.1. Materials

Broccoli (*Brassica oleracea* var. *italica*) was purchased from the local market (Melbourne, Australia). All chemicals used in the preparation and analysis of extracted cellulose, synthesized CMC, and preparation of CMC films were analytical reagent (AR) grade or the equivalent. Sodium hydroxide pellets, phenolphthalein, hydrogen peroxide solution, 30% (*w*/*w*), isopropyl alcohol, methanol, and sodium nitrite were delivered from Chem Supply Pty Ltd. (Adelaide, Australia). Chloroacetic acid was purchased from Sigma-Aldrich (St. Louis, Missouri, United States). Hydrochloric acid, 36% (*w*/*v*), silica gel, high vacuum grease, and glacial acetic acid were obtained from Thermo Fisher Scientific Inc. (Waltham, Massachusetts, United States). Glycerol and ethanol absolute were supplied from Merck Pty Ltd. (Burlington, Massachusetts, United States). Nitric acid (70%) was provided by RCI Labscan Ltd. (Rong Muang, Thailand). Sodium carboxymethyl cellulose powder was offered by Melbourne Food Ingredient Depot (Melbourne, Australia).

### 2.2. Material Preparation

Broccoli stems were cut off the florets and washed with tap water after removing the leaves from the stems. Aliquots of 800 grams of stems were cut into small pieces (~1 cm) and oven-dried at 90°C for 16 hours [21]. The dried stems were weighed to calculate the percentage of moisture content of fresh broccoli stems. The dried stems were grinded into powder using a grinder (Sunbeam, Boca Raton, Florida, United States) and stored in polypropylene bags at 4°C for further investigation.

### 2.3. Extraction of Cellulose

The different reaction parameters for cellulose extraction are presented in Table 1. The extraction of cellulose was divided into two stages: pulping and bleaching.

#### 2.3.1. Pulping

10 grams of dried stem powder were mixed with 1.25, 2.5, and 3.75 M NaOH solutions in a ratio of powder to solvent at 1 : 20 (*w*/*v*) and magnetically stirred at 100°C for 3 hr, 5 hr, and 7 hr. After that, the black slurry was vacuum suction filtered with 185 mm diameter filter paper and rinsed to remove lignin and hemicellulose.

#### 2.3.2. Bleaching

The cellulose powder was bleached with hydrogen peroxide (30%, *w*/*w*) for 3 hr at 70°C to completely remove lignin and hemicellulose. The obtained cellulose pulp was then vacuum-filtered, washed with distilled water, and oven-dried for 24 hr at 50°C [21, 27].

### 2.4. Yield of Cellulose

The yield of cellulose was expressed as the ratio of the mass of dried cellulose divided by the mass of initially dried broccoli stems as a percentage using the following equation:

(1) $$\begin{array}{c} \text{Yield of cellulose }\left(\%\right)=\frac{\text{weight of dried cellulose }\left(\text{g}\right)}{\text{weight of dried stem powder }\left(\text{g}\right)}\times 100\%. \end{array}$$

### 2.5. Synthesis of Carboxymethyl Cellulose (CMC)

The different reaction parameters for CMC synthesis are presented in Table 1. The synthesis of CMC was conducted in two steps: alkalization and etherification.

#### 2.5.1. Alkalization

Three grams of previously obtained cellulose were alkalized by dropping 2.5, 5, and 7.5 M NaOH solution under 60 mL of the support medium isopropanol. The mixture was vigorously stirred for 1 hr at ambient temperature.

#### 2.5.2. Etherification

After alkaline treatment, the etherification process was then carried out with the addition of chloroacetic acid (CAA). CAA was added to the alkali-treated mass with 1 : 1, 1 : 1.2, and 1 : 1.4 cellulose-to-CAA ratio and kept the reaction for 30 min, and then, the mixture was heated at 55°C and continuously stirred for 3 hr. The two-phase solution was vacuum filtered, and the residue was suspended in 100 mL methanol for 40 min. The suspended slurry was neutralized by glacial acetic acid several times to remove the excess alkali and vacuum-filtered again. The residue was then washed and filtered by 70% ethanol, followed by absolute methanol, and finally dried at 60°C overnight in an oven. The dried CMC powder was stored in polypropylene tubes at 4°C for further study [22, 24].

### 2.6. Characterization of CMC

#### 2.6.1. Yield of CMC

The yield of CMC, expressed as a percentage, was calculated based on the gravimetric method using the following equation:

(2) $$\begin{array}{c} \text{Yield of CMC }\left(\%\right)=\frac{\text{weight of CMC }\left(\text{g}\right)}{\text{weight of cellulose }\left(\text{g}\right)}\times 100\%. \end{array}$$

#### 2.6.2. Degree of Substitution (DS)

The degree of substitution (DS) is an important feature of CMC as it reflects the number of hydrogens substituted by carboxymethyl in the hydroxyl group of the cellulose glucose unit [28]. The absolute value of DS was performed by potentiometric back titration [23, 24]. 25 mL ethanol (95%) and 2.5 mL nitric acid (2 M) were added to 0.5 g CMC powder and thoroughly stirred for 10 minutes at room temperature, the mixed solution was heated to boiling for 5 minutes, and the heat was turned off and left to settle. After vacuum filtering the solution, the precipitate was rinsed with 100 mL of ethanol (95%) and methanol (100%) until all acids were removed. The residue was oven-dried for 3 hr at 105°C.

The CMC residue was weighed and mixed with distilled water (1 : 200 g/mL CMC to water ratio). NaOH solution (0.5 M) was added to the solution (1 : 50 g/mL CMC to NaOH ratio) and heated for 20 minutes. After cooling, the solution was titrated with 0.3 M HCl while observing the colour change from Mexican pink (dark pink) to colourless using phenolphthalein as an indicator. The absolute value of CMC's DS was calculated using the following equations:

(3) $$\begin{array}{c} A=\frac{BC-DE}{F}, \end{array}$$

(4) $$\begin{array}{c} \text{DS}=\frac{0.162\times A}{1-\left(0.058\times A\right)}, \end{array}$$

where *A* is the milliequivalents of consumed HCl per gram of specimen, *B* is the volume of NaOH added, *C* is the molarity of NaOH, *D* is the volume of consumed HCl, *E* is the molarity of HCl used, *F* is the CMC in grams, 162 is the molecular weight of the anhydrous glucose unit, and 58 is the net increment in the anhydrous glucose unit for every substituted carboxymethyl group.

#### 2.6.3. Viscosity

The viscosity of CMC powder was measured using an Analogue Brookfield Dial Reading viscometer (LV or RV) (Brookfield Engineering, Middleborough, Massachusetts, United States). Prior to analysis, 1 g/100 mL CMC solution was prepared. The viscosity was measured after CMC solution was cooled down to room temperature.

### 2.7. Preparation of CMC Films

CMC film preparation was carried out with some modifications reported by previous method [29]. The film-forming solutions were prepared by dissolving various concentrations of CMC powder in Milli-Q water (2, 4, and 6 g/100 mL), followed by the addition of glycerol (10%-30% (*w*/*w*) based on CMC, i.e., 0.2-1.8 g/100 mL) as a plasticizer (Table 1) and stirred constantly at 80°C for 20 minutes. After complete dissolution and dispersion, the mixed solutions were cast on a clean weighing plate (80 × 80 mm). The films were then oven-dried at 40°C overnight before being peeled off from the plates.

### 2.8. Characterization of CMC Films

#### 2.8.1. Tensile Strength (TS) and Elongation at Break (EB)

The tensile strength (TS) and percent elongation at break (EB) were determined from the stress-strain curves of the CMC films by the Instron 5944 2 kN Microtester (Instron, Coronation Road, High Wycombe, Bucks HP12 3SY, United Kingdom) according to the standard procedure of ASTM D882-02 [30]. The thickness of CMC film was determined using a digital vernier caliper.

Prior to testing, CMC films were preconditioned in a desiccator at room temperature (25°C) with 65% relative humidity for 24 hr, which was supported by saturated sodium nitrite solution. After that, CMC films were cut into 60 × 10 mm (length × width) strips by removing the uneven edge parts. Three specimens were studied for each film-forming case, with the initial grip separation being around 35 mm and the crosshead speed being 5 mm/min.

The TS and EB were determined by the following equations:

(5) $$\begin{array}{c} \text{Tensile strength }\left(\text{MPa}\right)=\frac{\text{the maximum load }\left(\text{N}\right)}{\text{width}\times \text{thickness of each film sample}}, \end{array}$$

(6) $$\begin{array}{c} \text{Elongation at break }\left(\%\right)=\frac{\text{the length of film rupture}-\text{the initial length of film sample}}{\text{the initial length of film sample}}. \end{array}$$

#### 2.8.2. Water Vapor Permeability (WVP)

The water vapor permeability (WVP) of CMC films was determined gravimetrically using the ASTM E96-95 standard method [31]. The test films were cut into discs slightly larger than the bottleneck's diameter of the experiment vials (1.2 cm diameter, containing blue silica gel). The film samples were then placed on top of the vials and sealed with high vacuum grease. The vials were placed in a desiccator (75% RH) and placed in a 25°C oven. Then, the vials were weighed daily over a seven-day period to provide the slope of weight gain (*Y* axis) and time (*X* axis). The water vapor transmission rate (WVTR) was calculated from the slope of the straight line divided by the test film area equation:

(7) $$\begin{array}{c} \text{WVTR}=\frac{\text{slope}}{\text{film area}}. \end{array}$$

Then, the water vapor permeability (WVP) was conducted by the following equation:

(8) $$\begin{array}{c} \text{WVP}=\frac{\text{WVTR}\times \text{thickness}}{\Delta P}, \end{array}$$

where Δ*P* is the partial water vapor pressure difference (mmHg) across the two sides of the film specimen (the vapor pressure of pure water at 25°C is 23.73 mmHg).

#### 2.8.3. Colour

The colour of CMC films was assessed using a chroma meter CR-400 (Konica Minolta, Marunouchi, Chiyoda, Tokyo, Japan) and expressed as three values: *L*^∗^ for lightness/blackness, *a*^∗^ for redness/greenness, and *b*^∗^ for yellowness/blueness. The reflection spectra of film samples placed on the surface of a standard white plate were used to obtain the results (*L*^∗^ = 92.66, *a*^∗^ = 0.41, *b*^∗^ = 0.03) [32]. Total colour differences (Δ*E*) account for comparisons between the *L*^∗^, *a*^∗^, and *b*^∗^ values of the sample and standard and are calculated using the following equation:

(9) $$\begin{array}{c} \Delta E=\sqrt{{\left(L_{\text{Standard}}^{*}-L_{\text{Sample}}^{*}\right)}^{2}+{\left(a_{\text{Standard}}^{*}-a_{\text{Sample}}^{*}\right)}^{2}+{\left(b_{\text{Standard}}^{*}-b_{\text{Sample}}^{*}\right)}^{2}.} \end{array}$$

#### 2.8.4. Film Thickness

The thickness of the film samples was measured at five random positions using a digital vernier caliper (Kincrome 3, Lakeview Dr, Caribbean Park, Scoresby, Victoria, Australia) with a precision of 0.01 mm.

### 2.9. Statistical Analysis

Data were analysed by one-way analysis of variance (ANOVA) and Tukey's test (*α* = 0.05) to assess significant differences (*p* < 0.05) using Microsoft Excel (Microsoft, Redmond, WA, USA) and conducted by XLSTAT ver. 2020.3.1 (Addinsoft, New York, NW, USA). All measurements were carried out in triplicate. The results represented the mean values ± standard deviation.

## 3. Results and Discussion

CMC was extracted from broccoli stem cellulose and was used to synthesize the biodegradable films. Characteristics of CMC powder and CMC films were investigated, and the results were shown below.

### 3.1. Extraction of Cellulose from Broccoli Stems

#### 3.1.1. Yield of Cellulose

**Figure 1:**
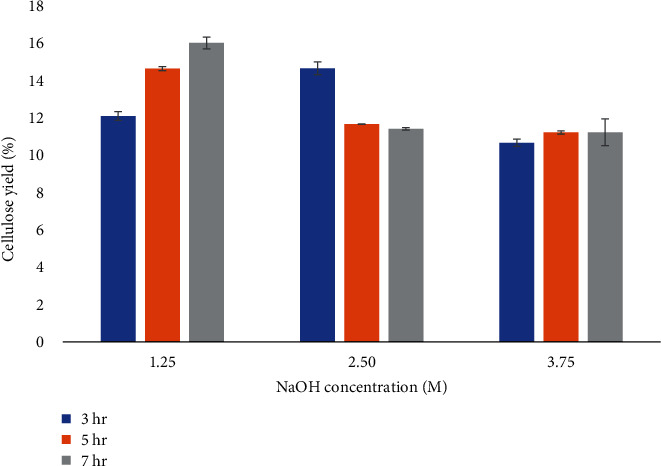
The yield of cellulose from broccoli stems at 100°C under 1.25, 2.5, and 3.75 M NaOH concentrations during 3, 5, and 7 hr. Values are given as mean ± standard deviation (*n* = 3).

The yield of cellulose was in the range of 10.66%-16.00% and cellulose extraction performed best with low NaOH concentrations and lengthy reaction duration.  Figure 1 displays the percentages of cellulose yield from broccoli stems under various NaOH concentrations (1.25-3.75 M) and 3-7 hr of reaction time.

At 1.25 M of NaOH, the cellulose yield increased significantly from 12.09% to 16.00% as the reaction time increased from 3 hr to 7 hr. In the alkaline treatment of cellulose, sodium (Na+) ions bring water close to the cellulose chain to first swell cellulose and remove the greater part of lignin and hemicellulose which are two main components accompanied cellulose [33]. This results in destroying the intermolecular bond between the ester and C-C bond, increasing the cellulose accessibility for further hydrolysis to small solvent molecules [34].

At higher concentration of NaOH (2.5 and 3.75 M), the cellulose yield decreased due to the alkali-catalyzed degradation of cellulose. This can be explained that if NaOH concentration is too high, the number of water molecules decreases to form solvated dipole hydrates; therefore, the hydration of alkali ions is insufficient to break hydrogen bonding [35].

It can be also explained that the cellulose and hemicelluloses are partly degraded and dissolved in an alkaline solution, which lead to decrease of yield and molecular weight of cellulose and hemicelluloses [36]. High NaOH concentration will also interrupt some of the crystalline region in cellulose and make cellulose easily dissolve in the solution treatment thus reduce yield of the cellulose fibre [37].

Similar outcomes were obtained from dried Mimosa pigra peel in which the percentage yield of cellulose increased as NaOH concentration (2.5-7.5 M) increased at 100°C for 3 hr, whereas it slightly decreased after increasing the concentration of NaOH above 7.5 M [29]. In another study, it was found that 4% NaOH gives the highest yield of cellulose from oil palm empty fruit bunch (OPEFB) and the yield decreased as the concentration of NaOH increased to 13% [37]. In extraction of cellulose from corncob, the maximum cellulose yield was obtained in an NaOH dosage of 50% (by dry corncob meter). Higher than this concentration, the cellulose yield decreased due to cellulose hydrolyzation [38].

In our study, the application of 1.25 M of NaOH increased the cellulose yield from 12.09% to 16.00% as the reaction time increased (3-7 hr; Figure 1). In contrast to the 2.5 M of NaOH extraction condition, cellulose yield declined when the heating time was increased from 3 hr to 5 hr. With the appropriate rise in alkali concentration and reaction duration, the chemical reaction will be fostered, and the delignification impact will be improved, which indicates higher yield of cellulose [37, 39]. Nevertheless, an excessive high concentration of NaOH or prolonged heating time will catalyze cellulose degradation, further resulting in a lower yield [37, 39].

The extraction methods of cellulose have been reported from many resources by different methods [10]. In most of these research, the optimum alkali solution heating parameters are in the ranges of 6–9% (*w*/*v*) NaOH, 3.5–5 hr, and 46–76°C, under continuous stirring. Therefore, optimizing the NaOH concentration, followed by the length of alkaline hydrolysis and the temperature, is an effective way to help mitigate alkali-catalyzed degradation and enhance cellulose yield. The results of our study are in agreements with these works. On the other hand, the application of complimentary methods such as ultrasound [40] and hydrogen peroxide under alkaline condition [41] is an effective way to increase the cellulose yield and reduce the further degradation of cellulose.

Alkaline pretreatment of lignocellulose biomass is a very popular and cost-effective process due to several advantages [42]. However, there are certain limitations related to the alkaline pretreatment of lignocellulose biomass. These include the nonselectivity of alkaline in removing lignin due to the loss of certain cellulose and hemicellulose components along with lignin. In addition, it requires the use of strong alkaline chemicals such as sodium hydroxide or ammonium hydroxide, the production, storage, and disposal of which may cause environmental issues [43].

The scalability and sustainability of caustic extraction of cellulose depend on a combination of efficient process design, responsible chemical usage, waste management practices, and technological advancements. Balancing the economic viability with environmental considerations is essential for the long-term application of the alkaline extraction of cellulose.

### 3.2. Synthesis of Carboxymethyl Cellulose from Broccoli Stems

#### 3.2.1. Yield of CMC

**Figure 2:**
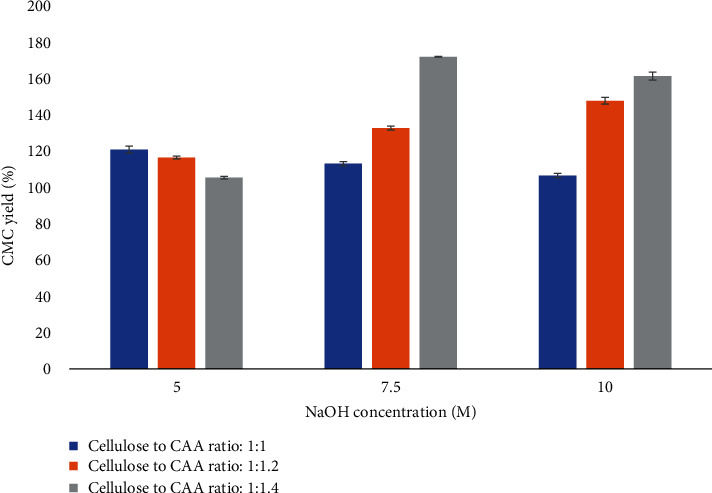
The yield of carboxymethyl cellulose under different NaOH concentrations (5-10 M) and C/CAA ratios (1 : 1-1 : 1.4). Values are given as mean ± standard deviation (*n* = 3).

The maximum yield of CMC (172.06%) was obtained at 7.5 M of NaOH and 1 : 1.2 cellulose-to-chloroacetic acid ratio (C/CAA). The percentages of carboxymethyl cellulose yield from broccoli cellulose under different NaOH concentrations and chloroacetic acid concentrations are shown in Figure 2.

At lower reagent addition (5 M NaOH), the CMC yield was at the minimum ranges and no significant difference between different C/CAA ratios on CMC yield was observed. It has been discussed that the low concentration of NaOH resulted in lower conversion of cellulose's hydroxyl group into reactive alkaline cellulose, which interferes with etherification and reduces yield [32].

The CMC yield increased from 110% to 170% by increasing C/CAA from 1 : 1 to 1 : 1.4 at 7.5 M of NaOH. Similar trends were also found at 10 M of NaOH concentration. High NaOH levels, such as 7.5 and 10 M, can change the crystalline region in cellulose to amorphous and thus, and it could be easily accessed by etherification agent (CAA). Lower ratio of CAA is not enough to etherify alkali cellulose to carboxymethyl cellulose. By increasing the concentration of CAA, the availability of acid molecules near the hydroxyl group of cellulose is increased as well, promoting the carboxymethylation process of cellulose [23, 44].

Nevertheless, lower reagent addition (5 M NaOH) tended to produce minimum CMC yield and had no significant difference between different C/CAA ratios on CMC yield results. It has been discussed that the low concentration of NaOH resulted in lower conversion of cellulose's hydroxyl group into reactive alkaline cellulose, which interferes with etherification and reduces yield [32]. High NaOH levels, such as 7.5 and 10 M, can successfully achieve the alkalization of cellulose, which contribute to more CMC yield. An increase in the C/CAA ratio also promotes the availability of acid molecules near the cellulose hydroxyls, facilitating carboxymethylation [23]. Our results confirm this response which aligns with results from asparagus stalks where it was observed that the yield of CMC increased with increasing NaOH concentrations (between 5 and 7.5 M), but after that point, the yield decreased with an increase in alkali concentration [22]. Rachtanapun et al. [32] also reported that 7.5-10 M of NaOH was a better condition for obtaining higher yield of CMC from durian rind, while the minimum percent yield of CMC was found under the use of 5 M of NaOH.

Factors affecting the robustness of cellulose extraction and CMC synthesis include the variations in raw material characteristics, such as the composition and properties of the source material (e.g., broccoli stems) and environmental conditions during cellulose extraction. In addition, the consistency of chemical reagents, reaction times, temperatures, and agitation during the synthesis process also plays a crucial role in determining reproducibility. Any deviations in these parameters may lead to variations in the final cellulose and CMC yields. Additionally, the purity of raw materials and the presence of impurities can affect the reproducibility of CMC synthesis [45].

#### 3.2.2. Degree of Substitution (DS) of CMC

**Figure 3:**
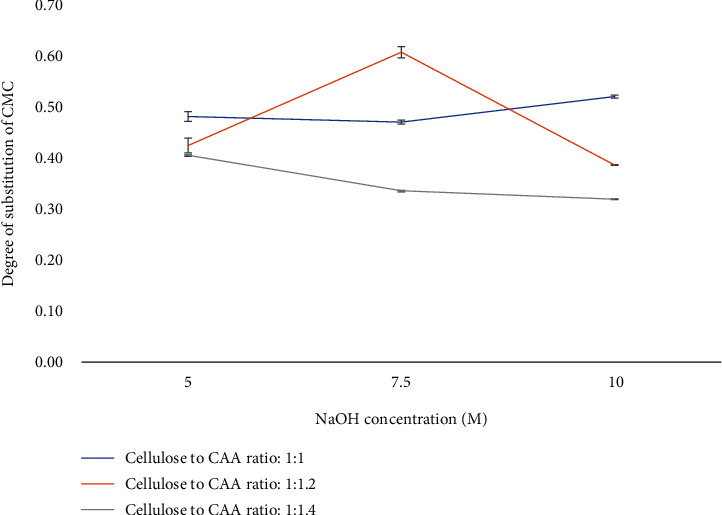
Degree of substitution (DS) of carboxymethyl cellulose under different NaOH concentrations (5-10 M) and C/CAA ratios (1 : 1-1 : 1.4). Values are given as mean ± standard deviation (*n* = 3).

The most critical characteristic of CMC is the degree of substitution and was determined by potentiometric back titration of our material [46]. The effects of reaction conditions, including the NaOH concentration and CAA concentration, on DS of CMC were studied (Figure 3). The CMC's DS value ranged from 0.32 to 0.66, with the highest value occurring at 7.5 M NaOH and a cellulose-to-CAA ratio of 1 : 1.2.

At 5 M of NaOH, the DS value of CMC decreased as more CAA was added. This may be due to the nonavailability of enough cellulose alkoxide for reaction with CAA. The excess CAA contributed to the formation of sodium glycolate and sodium chlorite in the side reaction of CAA with hydroxide ions [47]. The same trend was observed for DS values at 10 M of NAOH.

At 7.5 M of NaOH, DS increased with an increase in CAA from 1 to 1.2. This may be due to the greater availability of the acid molecules at higher concentrations in the proximity of the cellulose molecules. However, additional CAA led to decrease the DS significantly (*p* < 0.05), possibly due to competing reaction that might take place in excess amount of CAA. The side reaction of sodium glycolate production will dominate when the CAA concentration reaches a certain point, interfering with the CMC synthesis and lowering the reaction efficiency [24, 48]. The same trend was also discovered in sodium monochloroacetate (SMCA), the sodium salt form of CAA, which is another common etherifying agent. With the constant concentration of NaOH, DS of CMC increased from 0.452 to 1.07 by increasing the SMCA concentration (0.075 M-0.108 M) and further increase in the concentration of SMCA resulted a decrease in DS value. The higher concentration of etherifying agents means the greater availability of acetate ions which can react with cellulose. However, the efficiency of the reaction and the DS value of CMC will decrease when the addition amounts of etherifying agents exceed the ideal condition [23, 24]. Considering the two reaction variables, including the NAOH concentration and CAA, it was found that increase of DS was dependent significantly by increase of % NaOH than that of increase of CAA.

#### 3.2.3. Viscosity of CMC

The potential applications of CMC are dependent upon DS value, degree of purity, and rheological properties [10]. The rheological characteristics of CMC are known to be influenced by a variety of factors, including the concentration of CMC and the concentration of NaOH and CAA used to synthesize CMC [49].

These rheological properties of CMC are closely linked to its potential applications in food packaging. CMC's ability to form stable and viscoelastic films makes it suitable for coating and packaging materials. Its thickening properties are valuable for controlling the texture and stability of food products. Moreover, the film-forming ability of CMC contributes to its role in creating protective coatings for food packaging, enhancing shelf life, and providing a barrier against external factors [50].

**Figure 4:**
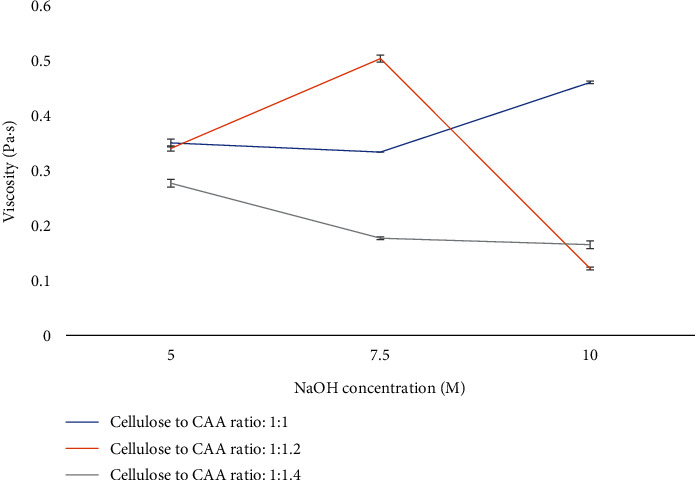
Viscosity (Pa·s) of 1 g/100 mL of carboxymethyl cellulose solution under different NaOH concentrations (5-10 M) and C/CAA ratios (1 : 1-1 : 1.4). Values are given as mean ± standard deviation (*n* = 3).

Viscosity is considered as an important parameter for assessing the rheological properties and has impact on CMC applications [23]. The conditions which produced the highest DS value of CMC (7.5 M of NaOH and C/CAA ratio at 1 : 1.2) resulted in the highest viscosity of 1 g/100 mL of CMC solution (0.5 Pa·s; Figure 4). DS value represents the degree of substitution of carboxymethyl groups which act as hydrophilic groups to immobilize water in the system. Generally, an increase in DS is associated with higher viscosity. This is because a higher DS means more carboxymethyl groups are present in the cellulose structure, leading to increased interaction and entanglement of polymer chains, resulting in higher viscosity [51].

Under C/CAA ratio at 1 : 1.2, the viscosity of CMC increased significantly (*p* < 0.05) from 0.34 to 0.5 Pa·s with increasing NaOH concentration from 5 to 7.5 M and then decreased when the NaOH concentration reached 10 M. This relationship was also observed for CMC extracted from durian rind [32], where the viscosity of CMC increased as the NaOH concentration increased from 5 to 7.5 M and then decreased with higher NaOH concentrations.

At a C/CAA ratio of 1 : 1, the viscosity of CMC significantly increased when NaOH reached 10 M, while it decreased significantly when the concentration of NaOH was above 5 M under C/CAA ratio at 1 : 1.4. This viscosity trend agrees with the DS value results previously mentioned in this experiment. In another attempt, the viscosity of CMC solution was improved by increasing the DS value of CMC extracted from Mimosa pigra peel [29]. The higher DS value represents the higher degree of substitution of carboxymethyl groups which act as hydrophilic groups to immobilize water in the system, which contributes to higher viscosity [32].

Practically, these viscosity changes have significant implications in real-world applications. In food products, CMC is often used as a thickening agent to control and enhance texture. The variation in DS allows for customization of viscosity, affecting the mouthfeel and overall sensory experience of food items. Furthermore, higher viscosity can contribute to stabilizing emulsions and suspensions in food formulations, preventing phase separation, and improving product quality.

In the context of food packaging applications, achieving a high degree of substitution (DS) in carboxymethyl cellulose (CMC) can have both advantages and potential trade-offs, particularly concerning viscosity. A higher DS often leads to improved film-forming capabilities of CMC. This is advantageous in food packaging as it allows for the creation of robust and effective protective coatings. On the other hand, achieving a very high DS might result in solutions that are too viscous for uniform application on packaging materials. This could lead to uneven coatings, affecting the overall effectiveness of the protective layer.

### 3.3. Characteristics of Carboxymethyl Cellulose Films

#### 3.3.1. Thickness of CMC Films

Film thickness may have impact on consumer acceptability. Consumers may associate film thickness with the perceived quality and robustness of the packaging. Thicker films may be perceived as more durable and protective. On the other side, excessively thin films might raise concerns about their strength and effectiveness, potentially affecting acceptability. Therefore, depending on the targeted product, the thickness should be modified.

The thickness of CMC film was affected by CMC concentration and plasticizer content. The thickness of CMC films prepared under different CMC concentrations with different glycerol concentrations is shown in Table 2. Films made of 6 g/100 mL of CMC were thicker than the other two concentrations of CMC solutions regardless of glycerol concentration (10-30% *w*/*w* based on CMC). The 2 and 4 g/100 mL of CMC solution did not result in significant differences in film thickness (*p* < 0.05). In addition, there was no correlation between the glycerol concentrations and CMC film thickness. This result was consistent with findings from a study working on CMC films made from seaweed waste with film-forming conditions of 0.23%-0.75% glycerol and 1%-3% (*w*/*v*) CMC concentrations [52] where CMC concentrations affected the biodegradable film thickness produced. The highest thickness value was 0.170 mm in a mixture of 0.75% glycerol and 3 g/100 mL CMC concentration, while the lowest thickness value was 0.107 mm in a mixture of 0.25% glycerol and 1 g/100 mL of CMC concentration. This phenomenon is considerably caused by CMC with more hydrophilic groups being better able to bind water and thus increase film thickness [18].

#### 3.3.2. Colour of CMC Films

The colour of CMC films was analysed to determine the effect of carboxymethylation reaction on CMC film's colour. The results were expressed as four values: *L*^∗^ for lightness/blackness, *a*^∗^ for redness/greenness, *b*^∗^ for yellowness/blueness, and *Δ*E for total colour difference (Table 3). There were significant differences (*p* < 0.05) between *b*^∗^ and Δ*E* among films created using 2 and 6 g/100 mL of CMC solution with glycerol addition (10-30% *w*/*w* based on CMC). The *a*^∗^ and Δ*E* were significantly different among films made of 4 g/100 mL CMC solution. The CMC films made of 6 g/100 mL of CMC solution had a higher *b*^∗^ values than the films prepared from 2 and 4 g/100 mL of CMC solution, indicating that the films tend to be more yellowish with the increase of CMC concentration. This might be the case because the CMC powder made from broccoli stems still had yellow undertones. It has been shown that as the NaOH concentration used for CMC synthesis increased, the yellowness of CMC film was further rose [21]. However, exceeding the NAOH level higher than a certain level led to decrease the yellowness of CMC films due to the reduction in carboxymethylation caused by the competitive reaction between NaOH and monochloroacetic acid [29]. In our study, there was not a systematic correlation between the colour of CMC films and carboxymethylation reaction.

The influence of CMC on broccoli on film yellowness is amplified in line with the increase in CMC concentration. Similarly, other studies have shown that films made of CMC from raw materials such as durian rind [32] and young palmyra palm fruit husk [21] exhibit more yellowness differences and are less transparent than those made of commercial CMC. Films with an observable yellow colour would be less acceptable for broad use as the colour will impact upon the perception of the wrapped product.

Adjusting reaction conditions, such as temperature, reaction time, and concentrations of reactants (NaOH and chloroacetic acid), can achieve colourless or minimally coloured CMC films suitable for use in food packaging. Optimizing these parameters helps to control the extent of carboxymethylation and minimize the formation of coloured by-products. Implementing effective purification processes, such as thorough washing and filtration steps, can help remove impurities and coloured by-products generated during the carboxymethylation reaction. This contributes to achieving a cleaner and more colourless CMC product.

#### 3.3.3. Mechanical Properties of CMC Films

Mechanical property is one of the most important factors for assessing the characteristics of films. A film's potential to be employed in the packaging industry is determined by its strong tensile strength, flexibility, and ductility. Tensile strength (TS) measures the stress required to break a material, while elongation at break (EB) is the corresponding strain at which the specimen fails [53]. These parameters are useful for the evaluation of the mechanical properties of film materials. For instance, a material with a combined high TS and high EB is indicative of high toughness, while materials with high TS and low EB tend to be brittle. Packaging material is required to sustain the integrity of the film to withstand extraneous forces. Mechanical properties such as tensile strength (TS) and elongation at break (EB) of packaging material are important to avoid the stress that develops during storage, processing, and handling.

In this research, tensile strength (TS) and elongation at break (EB) were two parameters to measure and demonstrate the films' mechanical properties. The effects of CMC's DS values and glycerol concentrations on the tensile strength and elongation after a break of CMC films are given in Table 4.

*(1) Tensile Strength*. The tensile strength (TS) of the broccoli-derived film was primarily related to the DS value of the CMC, and a higher DS value of CMC contributed to a stronger tensile strength (Table 4). Despite the different film-forming conditions, CMC with a DS of 0.60 can typically produce films with a good tensile strength. The primary reason is that participation in the carboxymethylation reaction results in an increase in substituted carboxymethyl groups in dehydrated glucopyranosyl units, which affects the increase in intermolecular forces and ionic properties between polymer chains, resulting in higher film strength [22]. This outcome is in line with the findings of Rodsamran and Sothornvit [54], who came to the conclusion that the DS value had an impact on the TS of films where the CMC film's TS (13.87 MPa) is higher than the CMC film's (3.20 MPa) because the DS value of CMC (0.75) is higher than that of CMC (0.64) synthesized from rice stubble. However, no correlation between the films' EB and DS values was noted. It might be that the crystallinity among CMC does not vary when DS value changes [21].

Regarding the effect of glycerol concentration on TS of CMC films, the CMC film made from 2, 4, and 6% CMC with a DS of 0.60 was studied since there was not a systematic correlation between the TS values in the other DS values.

At any CMC concentration with a DS value of 0.6, the TS value was firstly increased by increasing the glycerol concentration from 0.2 to 0.4 g/100 mL. This can be related to the antiplasticization phenomena that occur at relatively low plasticizer concentrations [55]. The antiplasticization effect is attributed to the association of glycerol with hydrophilic side groups, leading in a decrease in free volume in polymer matrix and suppressing the polymer chain motion and increasing the TS [56].

When the glycerol concentration increased up to 6%, the TS values for all CMC films at DS value of 0.6 decreased. This is due to the effect of glycerol in reducing the intramolecular bonds between the CMC chains as well as supporting the formation of H-bonds between glycerol and CMC molecules [57, 58].

*(2) Elongation at Break*. Elongation at break is a measure of the stretch ability of the film prior to breakage. The use of glycerol affected the EB of CMC films (Table 4). The EB values increased by increasing the glycerol concentration at any CMC concentration with a DS value of 0.6. This is due to this fact that glycerol reduced the direct intermolecular interactions and increased the free volume between the CMC chains and thereby increased the film flexibility [59, 60].

Glycerol content's impact on the mechanical characteristics of CMC films from Mimosa pigra peel was investigated, where the EB of CMC film increased from 15% to 25% with the increase in glycerol content from 10% to 30% and the TS of the film decreased from 10 MPa to 5 MPa [29]. This phenomenon is caused by high glycerol concentration that will break the hydrogen bond between adjacent molecules, lengthen the polymer chain, and increase the distance between molecules [18]. These consequences will increase the film's flexibility and elongation but will also decrease its mechanical resistance and lower its tensile strength, making it more likely to break under stress [57].

As two critical parameters commonly used to evaluate the mechanical properties of prepared films, the greater the maximum stress, the more the film can endure before breaking (TS), and the greater the shape change it can withstand without breaking (EB), the more suitable it is for use as a food packaging material.

The balance between flexibility and strength in CMC films is influenced by the concentration of glycerol. Lower glycerol concentrations strike a balance between flexibility and strength, making them suitable for applications where a certain degree of rigidity is desired alongside flexibility. Film thickness plays a role in determining the optimal glycerol concentration, with thicker films benefiting from higher concentrations to maintain flexibility, while thinner films may require lower concentrations to avoid overplasticization and potential reductions in strength. The intended use of CMC films guides the choice of glycerol concentration, with higher concentrations preferred for applications demanding high flexibility, such as in food packaging where conformability to the product is essential. Environmental conditions also come into play, as in humid environments, a higher glycerol concentration may be necessary to prevent the films from becoming too brittle. Overall, tailoring glycerol concentrations enables the optimization of CMC films for diverse applications, meeting specific requirements for flexibility and strength based on film thickness, intended use, and environmental factors.

Films with superior mechanical properties, such as strength, flexibility, and tear resistance, are more likely to be embraced by consumers. If CMC films offer equivalent or superior performance in terms of mechanical strength and flexibility, consumers may be more inclined to embrace them as a viable and sustainable alternative to traditional packaging materials.

#### 3.3.4. Water Vapor Permeability (WVP) of CMC Films

Water vapor permeability (WVP) is an important factor for films in food packaging applications [61]. Generally, the hydrophilicity or hydrophobicity of the polymer, which affects how the polymer interacts with water, has a significant impact on the WVP of films. Water would typically permeate the hydrophilic part of the film [62]. The water vapor permeability of CMC films made from 2, 4, and 6 g/100 mL of CMC solution with different DS values under 10-30% (*w*/*w* based on CMC) glycerol concentration was measured over a 7-day period (Table 4).

There were no significant differences between WVP values of the films and glycerol concentrations with the DS value of 0.6. The WVP of the films increased when the concentration of CMC film-forming solution increased from 2 to 6 (g/100 mL). It was found that 6 g/100 mL of CMC resulted in films with the highest WVP (7.87 × 10^3^ gm^2^·mmHg^−1^/day) compared to 2 and 4 g/100 mL of CMC film-forming solution at 30% of glycerol. The reason is mainly because the films made of higher concentration of CMC contain more carboxymethyl groups which are responsible for binding more water as mentioned before. As the concentration of CMC in a film increases, more hydrophilic sites become available for water absorption and diffusion through the film matrix, leading to an increase in WVP [32].

Moreover, the results indicated that the WVP of the films is associated with the DS value of the CMC. The films with the highest WVP (7.87 × 10^3^ gm^2^·mmHg^−1^/day) made with 6 g/100 mL of CMC had the highest DS value (0.60). This is due to the fact that WVP is a different characteristic that is directly related to the film's hydrophilicity, meaning that the film with a higher WVP will be more hydrophilic [30]. The increased DS value reflects the film's increased hydrophilicity and ease of water absorption by showing that more hydroxyl groups in CMC are being replaced by carboxymethyl groups.

Similar results have also been observed in films made from CMC synthesized from durian rind [32]. It was found that when the DS value of CMC increased from 0.56 to 0.87, WVTR of CMC films increased from 205 to 220 g/day·m^2^. Furthermore, Kamthai and Magaraphan [63] observed that films formed with CMC produced from bagasse pulp at higher alkaline concentrations, i.e., CMC with higher DS values, could allow more molecular movement to support water trans.

In food packaging applications, controlling moisture permeability is critical for preserving the quality, freshness, and shelf life of the packaged products. Higher WVP means that these films are more permeable to water vapor, potentially allowing moisture to pass through. The poor water vapor transmission properties of CMC films limit their application in industrial level. This problem can be overcome by incorporating some hydrophobic additives such as fatty acids into the cellulosic complex [64].

## 4. Conclusion

The current study makes noteworthy contributions in utilization of broccoli stems, typically discarded, by recognizing them as a valuable source for extracting cellulose. Additionally, the conversion of broccoli stems into CMC films aligns with waste reduction and recycling principles. Rather than disposing of these stems, a substantial portion of food waste, the research promotes repurposing them into functional materials, thereby diminishing the environmental repercussions of waste disposal.

The optimum condition for cellulose extraction from broccoli stems was obtained with the application of NaOH (1.25 M) with 7 hr heating time at 100°C, which resulted in 16% cellulose yield. It was found that the use of NaOH (7.5 M) and a 1 : 1.4 cellulose-to-chloroacetic acid ratio resulted in the highest yield of CMC (172.06%), while the conditions with the best CMC characteristics, such as the highest DS value of 0.60 and viscosity (0.5 Pa·s), were 7.5 M of NaOH and a 1 : 1.2 cellulose-to-chloroacetic acid ratio. The ideal conditions for CMC film formation vary depending on the properties required. The optimized mechanical properties were found for 4 g/100 mL of CMC concentration with 20% and 30% (*w*/*w* based on CMC) glycerol for tensile strength and elongation at break, respectively. 6 g/100 mL of CMC concentration was responsible for higher WVP of CMC films. Achieving the optimal balance in CMC film synthesis involves strategic considerations. Fine-tuning plasticizer concentrations, particularly glycerol, is crucial for ensuring flexibility without compromising strength or barrier properties. This delicate balance requires thoughtful assessment of the intended use and specific demands of the packaging application. Adjusting film structure and processing parameters, including casting conditions, and drying methods, also plays a key role in influencing both mechanical and barrier properties.

Efficient pretreatment technique to extract cellulose and transform it into CMC is a matter of concern because it involves the application of harsh chemicals, high temperatures, and high energy consumption leading to negative environmental impacts. Therefore, the green methods are offered as an alternative way to extract cellulose and produce CMC. These methods are assessed based on their effectiveness in delignification, decreased solvent necessity, environmental implications, economic feasibility, and alignment with recent advancements in sustainable pretreatment technologies [65].

The conversion of cellulose of broccoli stems to CMC and making biodegradable film can contribute to recycling food waste and loss. Carboxymethyl cellulose is a substance with a large range of applications, and there is more to be discovered. The discussion presented in this work can benefit the food industries such as food packaging to have an insight towards achieving sustainable food production and environmental protection. Further investigations are essential to acquire all influencing factors relating to the physical characteristics of CMC films as well as applying new technologies to extract cellulose from food waste materials.

## Figures and Tables

**Table 1:** Variables applied for cellulose extraction, CMC synthesis, and CMC film formation.

Extraction of cellulose	Concentration of NaOH (M)	Reaction time (hr)
Reaction temperature (°C)		

100	1.25	3
		5
		7
	2.5	3
		5
		7
	3.75	3
		5
		7

Synthesis of CMC		
Concentration of NaOH (M)	Cellulose-to-CAA ratio	

5	1 : 1	
	1 : 1.2	
	1 : 1.4	
7.5	1 : 1	
	1 : 1.2	
	1 : 1.4	
10	1 : 1	
	1 : 1.2	
	1 : 1.4	

CMC film formation		
Concentration of CMC (g/100 mL)	Glycerol addition (g/100 mL (10-30% *w*/*w* based on CMC))	

2	0.2	
	0.4	
	0.6	
4	0.4	
	0.8	
	1.2	
6	0.6	
	1.2	
	1.8	

**Table 2:** Thickness of CMC films made from 2, 4, and 6 g/100 mL CMC solution with glycerol (10-30% (*w*/*w* based on CMC)).

CMC concentration (g/100 mL)	Glycerol addition (g/100 mL (10-30% *w*/*w* based on CMC))	CMC films thickness (mm)
2	0.2	0.21 ± 0.02^ab^
	0.4	0.21 ± 0.02^b^
	0.6	0.23 ± 0.01^ab^

4	0.4	0.20 ± 0.01^b^
	0.8	0.24 ± 0.01^ab^
	1.2	0.21 ± 0.02^b^

6	0.6	0.30 ± 0.02^a^
	1.2	0.25 ± 0.02^ab^
	1.8	0.29 ± 0.02^a^

*Note*: values are given as mean ± standard deviation (*n* = 3). Different letters a and b in superscript interpreted significant differences (*p* < 0.05) between CMC films from different forming conditions.

**Table 3:** Colour of CMC films under different CMC concentrations (2, 4, and 6 g/100 mL) and glycerol (10-30% (*w*/*w* based on CMC)).

CMC (g/100 mL)	Glycerol (g/100 mL (10-30% *w*/*w* based on CMC))	NaOH for CMC synthesis (M)	*L* ^∗^	*a* ^∗^	*b* ^∗^	Δ*E*
2	0.2	5	86.71 ± 0.52^ab^	−2.05 ± 0.04^bc^	10.93 ± 0.57^abcde^	12.03 ± 0.01^fghi^
		7.5	72.74 ± 0.20^ab^	−0.98 ± 0.25^ab^	10.91 ± 0.11^abcde^	23.78 ± 0.08^bc^
		10	68.12 ± 0.31^ab^	−0.96 ± 0.02^ab^	5.29 ± 0.21^e^	25.10 ± 0.02^bc^
	0.4	5	88.71 ± 0.34^a^	−2.14 ± 0.04^bc^	10.08 ± 0.42^abcde^	11.36 ± 0.66^ghi^
		7.5	85.77 ± 0.54^ab^	−1.86 ± 0.08^abc^	13.56 ± 0.42^abcd^	15.30 ± 2.56^efg^
		10	89.05 ± 0.54^a^	−1.55 ± 0.07^abc^	6.11 ± 0.06^de^	7.02 ± 0.07^i^
	0.6	5	86.97 ± 0.34^ab^	−2.43 ± 0.03^bc^	12.26 ± 0.34^abcde^	13.66 ± 0.49^fgh^
		7.5	86.67 ± 0.34^ab^	−2.40 ± 0.10^bc^	12.47 ± 0.51^abcde^	13.98 ± 1.25^fgh^
		10	86.47 ± 0.30^ab^	−2.14 ± 0.02^bc^	9.28 ± 0.17^abcde^	11.26 ± 0.07^ghi^

4	0.4	5	80.22 ± 0.45^ab^	−2.01 ± 0.06^abc^	10.56 ± 0.50^abcde^	20.54 ± 0.22^cde^
		7.5	76.71 ± 0.56^ab^	−1.77 ± 0.04^abc^	10.68 ± 0.53^abcde^	20.81 ± 0.81^cde^
		10	88.13 ± 0.37^a^	−2.03 ± 0.13^abc^	8.27 ± 1.09^bcde^	9.54 ± 0.63^hi^
	0.8	5	89.75 ± 0.13^a^	−1.85 ± 0.04^abc^	7.03 ± 0.25^cde^	9.64 ± 0.58^hi^
		7.5	87.31 ± 0.21^ab^	−1.68 ± 0.25^abc^	13.71 ± 0.66^abc^	15.79 ± 0.06^defg^
		10	80.93 ± 0.30^ab^	−1.05 ± 0.01^abc^	9.15 ± 0.14^abcde^	14.87 ± 0.18^fgh^
	1.2	5	86.21 ± 0.37^ab^	−2.16 ± 0.04^bc^	9.72 ± 0.32^abcde^	13.31 ± 0.54^fgh^
		7.5	86.10 ± 0.33^ab^	−1.76 ± 0.02^abc^	9.88 ± 0.55^abcde^	15.84 ± 0.13^defg^
		10	63.00 ± 1.15^b^	−0.50 ± 0.31^a^	11.03 ± 0.92^abcde^	31.63 ± 0.04^a^

6	0.6	5	83.55 ± 0.43^ab^	−2.14 ± 0.06^bc^	12.44 ± 0.70^abcde^	15.50 ± 0.69^defg^
		7.5	78.42 ± 0.33^ab^	−1.95 ± 0.03^abc^	15.16 ± 0.56^ab^	21.37 ± 0.77^cd^
		10	84.77 ± 0.41^ab^	−1.34 ± 0.03^abc^	11.61 ± 0.77^abcde^	14.09 ± 0.27^fgh^
	1.2	5	84.83 ± 0.17^ab^	−2.28 ± 0.03^bc^	10.61 ± 0.11^abcde^	23.54 ± 0.19^bc^
		7.5	84.39 ± 0.21^ab^	−2.49 ± 0.04^bc^	15.10 ± 0.43^ab^	17.32 ± 1.98^def^
		10	70.97 ± 0.53^ab^	−2.35 ± 0.03^bc^	16.31 ± 0.13^a^	27.19 ± 0.34^ab^
	1.8	5	84.43 ± 0.11^ab^	−2.38 ± 0.01^bc^	13.96 ± 0.04^abc^	17.35 ± 0.79^def^
		7.5	85.05 ± 0.25^ab^	−2.26 ± 0.11^bc^	12.23 ± 0.77^abcde^	14.51 ± 0.51^fgh^
		10	71.60 ± 1.76^ab^	−2.57 ± 0.04^c^	12.47 ± 0.35^abcde^	24.54 ± 0.47^bc^

*Note*: values are given as mean ± standard deviation (*n* = 3). Different letters a-i in superscript interpreted significant differences (*p* < 0.05) between CMC films from different forming conditions.

**Table 4:** Mechanical properties of CMC films (DS value = 0.60) under different film-forming conditions (2-6 g/100 mL CMC with 10-30% (*w*/*w* based on CMC) glycerol).

CMC concentration (g/100 mL)	2			4			6		
Glycerol (g/100 mL (10-30% *w*/*w* based on CMC))	0.2	0.4	0.6	0.2	0.4	0.6	0.2	0.4	0.6
Tensile strength (MPa)	22.22 ± 2.32^ab^	28.02 ± 1.08^ab^	24.53 ± 0.01^ab^	25.77 ± 0.73^ab^	31.91 ± 5.90^a^	24.66 ± 1.46^ab^	22.01 ± 1.75^ab^	25.52 ± 1.41^ab^	18.69 ± 0.56^b^
Elongation at break (%)	0.52 ± 0.41^e^	13.2 ± 0.58^b^	22.93 ± 1.14^a^	1.74 ± 0.23^de^	7.23 ± 0.09^cd^	10.59 ± 0.00^bc^	6.86 ± 0.76^cd^	25.28 ± 2.67^a^	27.56 ± 0.09^a^
WVP (gm^2^·mmHg^−1^/day)	3.38 ± 0.07^cd^	2.05 ± 0.46^d^	5.16 ± 0.37^bc^	3.32 ± 0.07^bcd^	4.66 ± 0.01^bc^	3.61 ± 0.00^bcd^	5.92 ± 0.37^ab^	4.57 ± 0.09^bcd^	7.87 ± 0.63^a^

*Note:* values are given as mean ± standard deviation (*n* = 3). Different letters a-e in superscript interpreted significant differences (*p* < 0.05) between CMC films from different forming conditions.

## Data Availability

The research data used to support the findings of this study are available from the corresponding author upon request.
